# Influence of experience on dental implant placement: an in vitro comparison of freehand, static guided and dynamic navigation approaches

**DOI:** 10.1186/s40729-022-00441-3

**Published:** 2022-10-10

**Authors:** Xiaotong Wang, Eman Shaheen, Sohaib Shujaat, Jan Meeus, Paul Legrand, Pierre Lahoud, Maurício do Nascimento Gerhardt, Constantinus Politis, Reinhilde Jacobs

**Affiliations:** 1 OMFS IMPATH Research Group, Department of Imaging and Pathology, KU Leuven, Kapucijnenvoer 33, 3000 Leuven, Belgium; 2 Department of Oral and Maxillofacial Surgery, The First Affiliated Hospital of Harbin Medical University, Youzheng Street 23, NangangHarbin, 150001 China; 3 Department of Oral and Maxillofacial Surgery, University Hospitals Leuven, Kapucijnenvoer 33, 3000 Leuven, Belgium; 4 Department of Maxillofacial Surgery and Diagnostic Sciences, College of Dentistry, King Saud Bin Abdulaziz University for Health Sciences, Riyadh, 14611 Saudi Arabia; 5 School of Health Sciences, Faculty of Dentistry, Pontifical Catholic University of Rio Grande Do Sul, Porto Alegre, 90619-900 Brazil; 6 Department of Dental Medicine, Karolinska Institutet, Alfred Nobels allé 8, 141 52 Huddinge, Sweden

**Keywords:** Dental implant, Surgical guide, Dynamic navigation, Dental education

## Abstract

**Purpose:**

This study aimed to investigate the performance of novice versus experienced practitioners for placing dental implant using freehand, static guided and dynamic navigation approaches.

**Methods:**

A total of 72 implants were placed in 36 simulation models. Three experienced and three novice practitioners were recruited for performing the osteotomy and implant insertion with freehand, surgical guide (pilot-drill guidance) and navigation (X-Guide, X-Nav technologies) approaches. Each practitioner inserted 4 implants per approach randomly with a 1-week gap to avoid memory bias (4 insertion sites × 3 approaches × 6 practitioners = 72 implants). The performance of practitioners was assessed by comparing actual implant deviation to the planned position, time required for implant placement and questionnaire-based self-confidence evaluation of practitioners on a scale of 1–30.

**Results:**

The navigation approach significantly improved angular deviation compared with freehand (*P* < 0.001) and surgical guide (*P* < 0.001) irrespective of the experience. Surgical time with navigation was significantly longer compared to the freehand approach (*P* < 0.001), where experienced practitioners performed significantly faster compared to novice practitioners (*P* < 0.001). Overall, self-confidence was higher in favor of novice practitioners with both guided approaches. In addition, the confidence of novice practitioners (median score = 26) was comparable to that of experienced practitioners (median score = 27) for placing implants with the navigation approach.

**Conclusions:**

Dynamic navigation system could act as a viable tool for dental implant placement. Unlike freehand and static-guided approaches, novice practitioners showed comparable accuracy and self-confidence to that of experienced practitioners with the navigation approach.

**Graphical Abstract:**

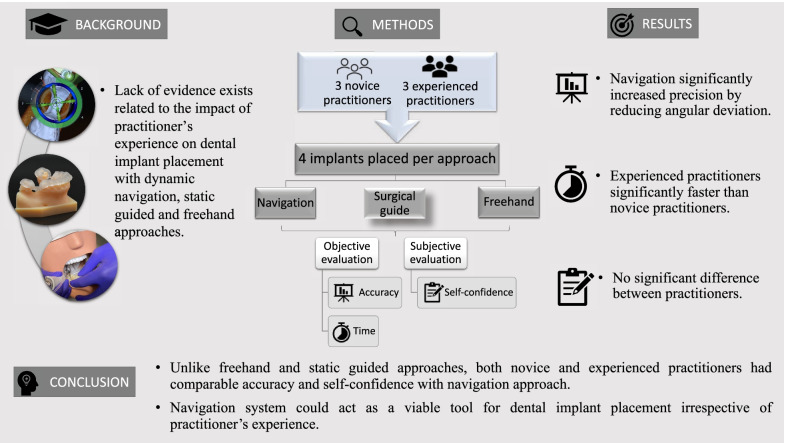

**Supplementary Information:**

The online version contains supplementary material available at 10.1186/s40729-022-00441-3.

## Introduction

Dental implant surgery has become a common practice for novice dental practitioners, which was once considered only under the domain of implant specialists and consultants. With its growing popularity for oral rehabilitation, the demand for clinical training has also increased [1]. A practitioner must be well-acquainted with the procedure and should have sufficient training for delivering a successful surgical and restorative outcome. However, most practitioners have limited surgical training which could increase the risk of inaccurate implant placement and complication rate [2]. In addition, one of the main challenges observed by novice practitioners is the optimal controlling of surgical osteotomy and implant positioning. A non-ideal implant placement makes the restoration far more difficult with the possibility of increased cost and time [3].

Recently, the application of cone-beam computed tomographic (CBCT) imaging and virtual planning software programs have facilitated accurate implant placement with a relative reduction in intraoperative complications [4–6]. Furthermore, the development of computer-guided surgical techniques, including static and dynamic approaches have improved the performance of novice practitioners and made it possible to transfer the planned implant position to the surgical site with a higher precision and less observer variability compared to conventional freehand technique [7, 8].

The commonly applied static guided techniques for implant placement involve either a pilot drill guided approach (only guided pilot osteotomy followed by freehand osteotomy and implant placement) or a fully guided approach (fully guided osteotomy and implant placement) [9]. In general, a static fully guided approach offers less deviation compared to a pilot-drill guidance; however, both approaches are considered clinically acceptable [10]. Nevertheless, pilot-drill guidance is a more simplified and commonly applied technique in a clinical setting with added advantages of controlled irrigation, easy access in patients with limited mouth opening and ability to manually adjust implant position or angulation [10]. In contrast to static approaches, the dynamic navigation systems have further improved the precision of the implant placement procedure which offer a real-time tracking of the drills and implant in accordance with the virtual planning [4, 7, 11].

Previous studies have reported that novice practitioners offer an improved level of accuracy for implant placement with lesser deviation with both static and dynamic guided approaches [5, 10, 12, 13]. However, lack of evidence exists related to the assessment of the accuracy and efficacy of novice compared to experienced practitioners for dental implant placement with freehand and guided approaches. Therefore, the primary aim of this in-vitro study was to evaluate the influence of practitioner’s experience on the accuracy of dental implant placement using freehand, static guided and dynamic navigation approaches. The secondary aims were to assess the surgical timing and self-confidence of practitioners. The null hypothesis was that no significant differences would exist between novice and experienced practitioners for implant placement with freehand, static guided and dynamic navigation approaches in relation to accuracy, surgical timing and self-confidence.

## Materials and methods

### Study sample

This research was performed in compliance with the World Medical Association Declaration of Helsinki on medical research. The study was approved by the Ethical Review Board of the University Hospitals Leuven, Belgium (reference number: S64493).

Dental implants were placed using three surgical approaches, i.e., freehand, surgical guide (pilot-drill guidance) and navigation system (Dynamic Navigation system, X-Guide, X-Nav technologies, LLC, Lansdale, PA). Sample size was calculated in G*Power v.3.1 (Heinrich-Heine Universität, Düsseldorf, Germany) with the following parameters: angular deviation data extracted from a study as the primary outcome variable [14] with alpha level of 0.05, statistical power of 80%, and effect size of 0.08 [15]. The calculation resulted in a total sample size of 36 implants required for the comparison of three approaches (*n* = 12 per approach).

A mandibular CBCT image having missing bilateral first molars (Fédération Dentaire Internationale [FDI], lower left 1st molar: 36, lower right 1st molar: 46) was retrospectively recruited from a radiological database. The scanning parameters were 110 kV, 8 × 10-cm field of view (FOV), and voxel size of 0.25 mm. Volumetric reconstruction of the mandibular bone was performed in Mimics software (version 21.0, Materialise, Leuven, Belgium). Thereafter, 36 identical simulation models were fabricated using Objet Connex 350 printer (Stratasys, Eden Prairie, MN, USA) with an acrylic-based resin (VeroDent MED670, Stratasys, Eden Prairie, MN, USA) [16].

Three experienced and three novice practitioners were recruited. Experienced practitioners consisted of oral surgeons with a clinical experience of over 5 years in implant dentistry and novice practitioners were general dentists with no clinical experience in implant dentistry. Prior to research, all practitioners received standard hands-on training for virtual planning with implant treatment planning software (DTX Studio™ Implant 3.4.3.3, Nobel Biocare AG) and surgical procedure simulation with the navigation system to achieve minimal proficiency. In addition, novice practitioners were also trained by an experienced clinician for performing implant placement with surgical guide and freehand approaches.

### Treatment planning

The planning for static-guide-based implant placement was performed using an open-source implant planning software (Blue Sky Plan 4, Blue Sky Bio LLC, Grayslake, IL, USA), where CBCT and intraoral scanned (IOS) images of the teeth were imported and registered. As the teeth derived from CBCT data set fail to display teeth accurately, the integration of intraoral scanned image through the registration step allowed to achieve precise occlusal surface details for the construction of a properly fitting surgical guide. Following virtual implant placement, a surgical guide was designed and exported in standard tessellation language (STL) format. The guide was printed using Objet Connex 350 printer with a polyjet material (MED610, Stratasys, Eden Prairie, MN, USA) and surgical sleeves were fixed onto the guide with an adhesive.

For navigation-based planning, a tracking device (X-Clip, X-Nav Technologies) with 3 radiopaque fiducials was fixed to the mandibular anterior and premolar teeth with a thermoplastic impression material. The acquired impression surface was printed with a soft transparent material (Tango +, Stratasys, Eden Prairie, MN, USA) which was then used to fix the X-clip with the teeth. This allowed replication of the registration with exact seating of the device onto the teeth of each model. A CBCT scan (Accuitomo*,* J. Morita, Kyoto, Japan) of the model with the adapted clip was acquired with the following acquisition parameters: 90 kV, 5 mA, full-scan mode (360°) with Hi-Fi*,* 0.125 mm voxel size and 8 × 8 cm FOV.

**Fig. 1:**
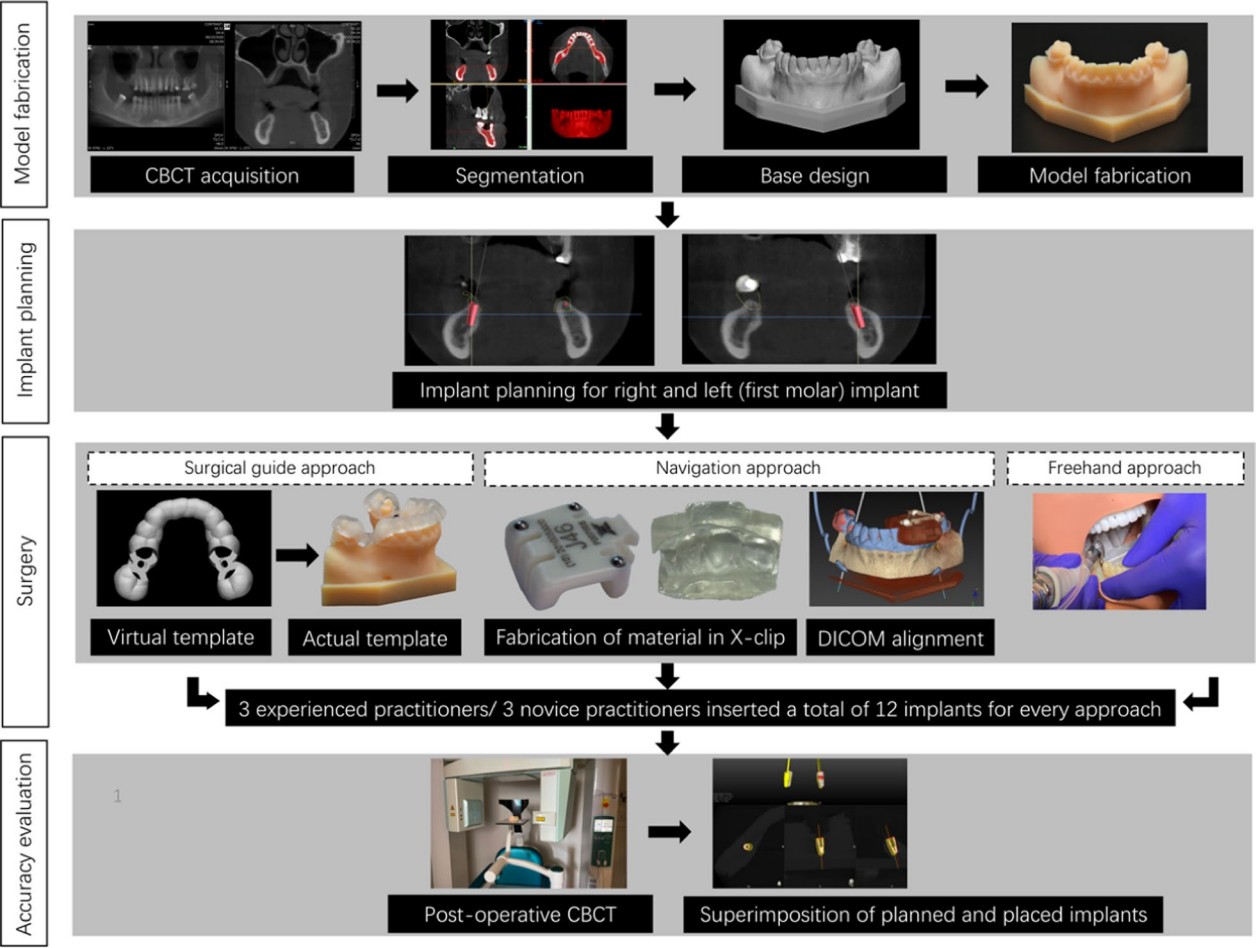
Workflow for surgery

The CBCT images of both patient and model were imported to Mimics Innovation Suite (Materialise, Leuven, Belgium) in Digital Imaging and Communications in Medicine (DICOM) format for aligning and combining the two images. This combined DICOM data set and IOS image of the teeth were uploaded and registered in DTX Studio implant software. The implants were virtually positioned at 36 and 46 sites similar to the static guide-based planning. Thereafter, all the images and virtual planning were transferred to the navigation system. Figure 1 represents the workflow for the surgery.

### Research procedure

All the practitioners were assigned with the task of inserting implants by each approach. The approach order was randomized for each practitioner using the random function of Microsoft Excel (version 16.38, Microsoft Corp, Redmond, US) and a 1-week gap was applied in-between approaches to avoid memory bias. The surgical procedure was standardized beforehand and the drilling sequence was prepared with irrigation based on a protocol recommended by the manufacturer (Wego, China). Following osteotomy, implants (customized experimental In-Hex implant, 3.8 mm × 9 mm, Wego, China) were placed using a surgical motor (EXPERTsurg™ LUX, KaVo, Germany) at 15 rpm and with a maximum torque of 50 N.cm.

**Fig. 2:**
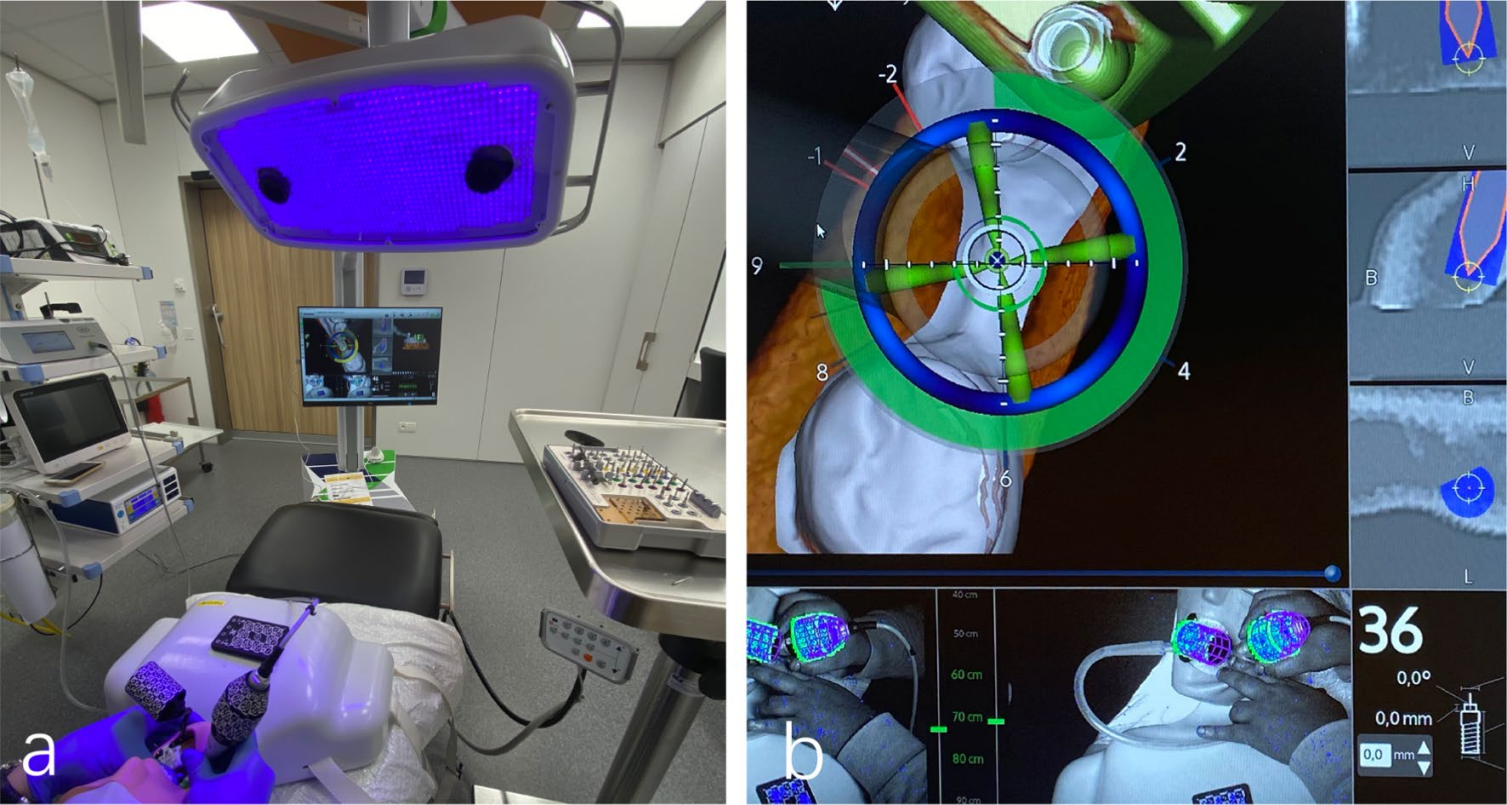
Navigation surgery. **a** Navigation system overview. **b** Screen displays implant site preparation

Each model was fixed onto a dental phantom head (Frasaco GmbH, Tettnang, Germany) for mimicking a clinical scenario (Fig. 2a). For the freehand approach, the practitioners used the planned implant position displayed on the Blue Sky Plan software as a reference. The static guide-based approach involved pilot drill guided osteotomy followed by freehand osteotomy and implant insertion.

The navigation-based approach involved rigid fixation of the X-clip onto the teeth with the printed impression surface of teeth. A rigid fixation allowed to keep the clip stable, as any clip movement and its instability during the procedure could induce error in the registration and calibration process of the navigation system. Hence, directly impact the accuracy of implant placement. The calibration of tracking arrays and handpiece were performed with a calibrating plate for verifying any deviation prior to the surgery. Both the drills and implant were tracked live by the system during insertion and the practitioners followed the planned path as displayed on the screen (Fig. 2b).

A post-operative CBCT scan of the drilled models was acquired using prior acquisition parameters. Thereafter, both pre-operative and post-operative CBCT images were superimposed to assess the deviation between planned and actual implant placement automatically with EvaluNav software (ClaroNav Technology Inc., Toronto, Canada). The parameters for assessing deviation included:

i) entry two-dimensional (2D) deviation (horizontal drilling point deviation),
ii) apex three-dimensional (3D) deviation (3D deviation at implant’s apex location),
iii) apex (V) deviation (vertical depth deviation)
iv) angular deviation.

The surgical time was recorded. In addition, a validated self-confidence questionnaire was conducted for evaluating the self-efficacy of practitioners on a scale of 1–30 for each approach (Additional file 1: Table S1) [17].

### Statistical analysis

Data were analyzed using IBM SPSS Statistics for Windows, version 21.0 (IBM Corp., Armonk, NY, USA). Descriptive statistics for all the parameters were recorded (entry 2D, apex 3D, apex V, angulation and surgical time). The Shapiro–Wilk test was used to test the normality of data distribution and data transformation was applied if required to adjust for the lack of normality. A linear mixed model with two fixed factors (experience and approach) and two random factors (surgeon and 3D printed model) was applied to examine the differences between each approach. A *P* value of < 0.05 was considered as statistically significant.

## Results

A total of 72 implants (4 insertion sites × 3 approaches × 6 practitioners = 72 implants) were placed by three experienced (12 implants per practitioner = 36 implants) and three novice practitioners (12 implants per practitioner = 36 implants). Two implant sites suffered from perforation at the apical part of lingual bone following drilling with freehand approach by experienced practitioners, while novice practitioners perforated lingual bone at two sites using surgical guide. In addition, a guide was fractured by a novice practitioner during osteotomy.

Table 1 describes the mean deviation between planned and actual implant position and time taken by each approach. In addition, the statistical significance of implant deviation, time and self-confidence based on approach, experience, and interaction of both is presented in Table 2. Following verification of residual values normality in the transformed data, the linear mixed model showed that the navigation approach significantly improved angular deviation compared with freehand (*P* < 0.001) and surgical guide (*P* < 0.001). Furthermore, experienced practitioners showed a slightly higher angular deviation with all three approaches compared to novice practitioners without any significant difference. The differences in entry 2D, apex 3D and apex V were not significantly different based on approaches, experience or interaction of both (*P* > 0.05).

**Table 1:** Descriptive values (Mean ± SD, Range) categorized by surgical approach and experience

Approach	Entry/mm	Apex(3D)/mm	Apex(V)/mm	Angle/°	Time/sec
Freehand					
Experienced	1.11 ± 0.58 (0.29–2.34)	1.91 ± 1.06 (0.97–3.93)	0.54 ± 0.38 (0.1–1.2)	9.73 ± 4.29 (4.01–17.65)	3.27 ± 1.43 (2.12–6.67)
Novice	1.40 ± 1.01 (0.09–3.15)	2.54 ± 1.58 (0.85–6.33)	0.60 ± 0.33 (0.1–1.09)	8.15 ± 4.73 (3.37–21.28)	7.33 ± 3.40 (3.25–13.17)
Surgical guide					
Experienced	0.83 ± 0.65 (0.1–2.24)	1.67 ± 0.94 (0.38–3.64)	0.48 ± 0.34 (0.01–0.97)	7.27 ± 3.82 (1.5–13.89)	3.62 ± 1.78 (1.62–7.65)
Novice	0.92 ± 0.38 (0.31–1.58)	1.66 ± 0.64 (0.48–2.75)	0.41 ± 0.27 (0.03–0.89)	7.07 ± 4.38 (1.45–15.36)	7.59 ± 2.17 (4.48–11.28)
Navigation					
Experienced	1.09 ± 0.41 (0.37–1.67)	1.55 ± 0.56 (0.65–2.77)	0.44 ± 0.55 (0.04–1.96)	3.37 ± 1.56 (1.61–6.68)	11.58 ± 3.51 (6.77–19.03)
Novice	1.14 ± 0.46 (0.4–2.02)	1.76 ± 0.71 (0.81–2.75)	0.70 ± 0.58 (0.14–2.2)	3.19 ± 1.89 (1.25–6.54)	13.08 ± 4.62 (5.75–20.33)

**Table 2:** Statistical significance of implant deviation, time and self-confidence considering approach, experience, and interaction of both

	Approach	Experience	Approach × Experience
Entry/mm	0.67	0.28	0.88
Apex(3d)/mm	0.15	0.19	0.78
Apex(v)/mm	0.39	0.23	0.41
Angle/°	**< 0.001**	0.35	0.84
Time/sec	**< 0.001**	**< 0.001**	**0.001**
Self-confidence	0.48	0.63	0.56

Numbers in bold refer to statistically significant values

The surgical time with navigation approach was significantly longer than that of freehand (*P* < 0.001) and surgical guide (*P* < 0.001). In addition, novice practitioners showed an overall increase in surgical time compared with experienced practitioners (*P* < 0.001). A significant difference in interaction was observed, which indicated that both experience and approach affected the surgical time (*P* = 0.001). The time taken by novice practitioners with navigation approach was significantly longer compared to experienced practitioners.

**Fig. 3:**
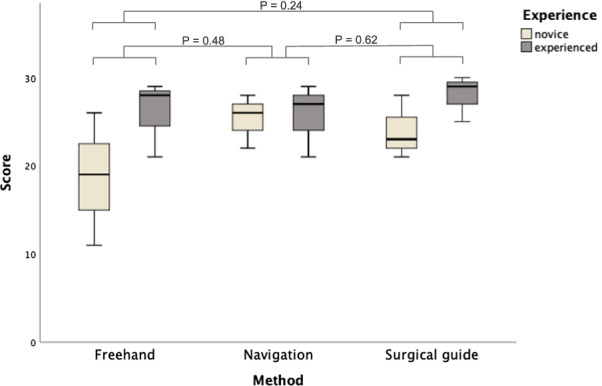
Median and inter-quartile range of self-confidence scores for each approach categorized by experience. Boxes comprise of 25th and 75th quartiles and median values, upper and lower whisker indicate highest and lowest values

The findings of the self-confidence questionnaire (Table 3) suggested no significant difference between self-confidence of both novice and experienced practitioners. However, novice practitioners considered that their performance improved using both guided approaches (Fig. 3), where they showed high level of confidence and lower anxiety with both guided approaches compared to the freehand approach. The scoring of novice practitioners’ self-confidence with the navigation approach (median score = 26) was comparable to that of experienced ones (median score = 27). In addition, experienced practitioners reported highest self-confidence scores with static guide (median score = 29), followed by freehand (median score = 28) and navigation system (median score = 27).

**Table 3:** Self-confidence scoring of each practitioner

		1. How confident were you during the procedure?	2. What was your surgical skill level during the procedure?	3. Were you worried during the procedure?	4. Were you anxious during the procedure?	5. Based on your performance today, would you have liked to have avoided this procedure altogether?	6. How comfortable were you with the independent planning and performing the procedure?	Total
Freehand	Experienced 1	4	5	5	4	5	5	28
	Experienced 2	4	4	3	3	4	3	21
	Experienced 3	5	5	5	5	5	4	29
	Novice 1	1	2	2	2	3	1	11
	Novice 2	2	3	3	3	5	3	19
	Novice 3	4	4	4	5	4	5	26
Surgical guide	Experienced 1	5	4	5	5	5	5	29
	Experienced 2	5	5	4	3	4	4	25
	Experienced 3	5	5	5	5	5	5	30
	Novice 1	3	3	3	3	5	4	21
	Novice 2	5	4	5	5	5	4	28
	Novice 3	3	3	4	5	3	5	23
Navigation	Experienced 1	4	5	5	4	5	4	27
	Experienced 2	5	5	5	4	5	5	29
	Experienced 3	3	5	3	3	4	3	21
	Novice 1	4	4	5	4	5	4	26
	Novice 2	3	4	3	4	4	4	22
	Novice 3	5	5	5	5	3	5	28

## Discussion

The implementation of computer-guided technologies promise a novel approach for dental implant surgery. This study investigated the accuracy, time-efficiency and self-confidence of novice practitioners compared to experienced practitioners for implant placement with freehand, static pilot drill-based guidance and navigation approaches. The findings suggested that the navigation approach could acted as a viable medium for performing implant surgery by novice practitioners with comparable accuracy, self-confidence and surgical time to that of experienced practitioners with the same level of training.

The angular deviation of implant placement was significantly better with navigation compared to freehand and surgical guided approach. As the freehand drilling mainly depends on the practitioner’s theoretical and clinical skills which are often acquired over a long period of time during training; therefore, it was difficult for novice practitioners to place the implant in an ideal position. In addition, posterior implant placements are generally less accurate than anterior ones owing to difficult access and indirect visualization which might have further contributed toward lower accuracy with the freehand approach [18]. In contrast, pilot-drill guidance offered the advantage of improved implant deviation compared to the freehand approach. However, it was still prone to a large angular deviation which could have resulted due to an undesirable mechanical tolerance between the drills and sleeve or accumulative error at the data acquisition, software processing and template manufacturing steps of the digital workflow [19]. In addition, the findings suggested that experienced practitioners offered higher angular deviation compared to novice practitioners with all the approaches. However, the difference was quite minimal which is negligible from a clinical point of view and could be attributed to the small sample of practitioners. Another reason could be related to the level of attention to detail and concentration, where novice practitioners might have paid more attention to avoid any unnecessary change in angulation.

The navigation approach provided the most accurate approach for implant placement with an excellent performance by novice practitioners. These findings were consistent with previous studies, where the navigation system offered significant improvement in implant placement accuracy compared to surgical guide and freehand approach [14, 20, 21]. At the same instance, a risk of implant deviation still exists with the navigation system due to the errors generated during the workflow steps of image acquisition, tracking clip stability, registration and calibration [14]. A practitioner should be aware of these errors which is crucial for a successful treatment outcome. However, the navigation approach allowed novice practitioners to achieve similar accuracy to that of experienced ones which was in accordance with another study [13]. Similarly, Sun et al. and Wu et al. found that the experience level of practitioners did not affect the accuracy of implant placement with the navigation approach [22, 23].

The surgical time required by the navigation approach was significantly longer than the surgical guide or freehand approach which was consistent with a previous study [15]. This increased time was attributed to the necessary calibration of the drills and implant required throughout the procedure to allow for optimal tracking. In addition, reconfirmation of the correct drilling and implant placement by viewing both the screen and patient led to a further increase in time. It should be noted that the navigation system has a steep learning curve, where its more frequent usage could allow mastering the system with a higher confidence in the technology and further lower the surgical time [24, 25]. At the same instance, surgical time with the navigation system could be less compared to other approaches in complex surgical cases with limited direct access or tight interdental spaces which preclude the usage of surgical guide tubes [8, 15].

Although the dynamic navigation system offers comparable accuracy to a static guided approach, its application is limited in a clinical practice due to high costs, steep learning curve, early developmental stage and risk of inaccurate implant placement due to system error associated with either registration or calibration steps especially in completely edentulous cases [26]. Furthermore, the majority of evidence assessing the accuracy of navigation systems is based on in vitro studies and clinical studies are still scarce. Hence, further clinical studies are required to confirm whether their implant positional accuracy and time efficiency is maintained in a real clinical scenario, where different patient- and surgery-related factors could negatively impact the final outcome. In contrast, ample evidence exists justifying the satisfactory results of static guided approaches in both partial and complete edentulous cases with a relatively lower price tag [6].

In the present study, novice practitioners required more time to perform the surgery irrespective of the approach, which could be attributed to the proficiency and surgical skills of the practitioners. The self-confidence of novice practitioners was high with both guided approaches, which was partially consistent with another study, where observers showed better performance and high confidence with a static guided approach [27, 28]. Furthermore, the novice practitioners expressed a desire to use the navigation system for future implantations which was consistent with previous studies [24, 29]. Experienced practitioners were more confident with a static guided approach compared to navigation system as they preferred relying on already achieved skills rather than pursuing new innovative technologies with complex workflows [30]. Hence, their performance with static guide was more predictable and less stressful which was confirmed by a higher self-confidence score.

The study had certain limitations. First, the findings of this study should be interpreted with caution due to its in-vitro study design. Second, a lack of variability existed in relation to implant insertion sites with only involvement of posterior region. Third, the study only assessed pilot-drill guidance. Hence, further studies are required to investigate the practitioner’s performance based on a static fully guided approach and with the inclusion of variable implant insertion sites.

## Conclusions

The dynamic navigation system could act as a viable tool for dental implant placement by novice practitioners, who were able to achieve comparable accuracy and self-confidence to that of experienced practitioners. The navigation approach offered a more accurate implant placement with significant improvement in angular deviation compared to the surgical guide and freehand approach irrespective of practitioner’s experience. Future clinical studies are required for the assessment of external validity and implant placement accuracy with navigation system in a clinical practice.

## Supplementary Information

**Additional file 1:** Questionnaire for evaluating practitioner’s self-confidence.

## Data Availability

The data sets used and/or analyzed during the current study are available from the corresponding author on reasonable request.

## Abbreviations

2D: Two-dimensional
3D: Three-dimensional
CBCT: Cone-beam computed tomography
DICOM: Digital Imaging and Communications in Medicine
FOV: Field of view
IOS: Intraoral scanned
STL: Standard tessellation language
V: Vertical
