# Supplementary material for: Influence of experience on dental implant placement: an in vitro comparison of freehand, static guided and dynamic navigation approaches
Source: Int J Implant Dent. 2022 Oct 10;8:42. doi: 10.1186/s40729-022-00441-3 (PMC9548458; doi:10.1186/s40729-022-00441-3)
Supplement: Supplementary file 1 — Additional file 1: Questionnaire for evaluating practitioner’s self-confidence. [file 40729_2022_441_MOESM1_ESM.docx]

Supplementary Table 1. Questionnaire for evaluating practitioner’s self-confidence.

**Please rate your performance on the procedure you just performed:**

**1. How confident were you during the procedure? Total Score: /30**

| **1** | **2** | **3** | **4** | **5** |
| --- | --- | --- | --- | --- |
| Not at all |  | Confident |  | Very confident, level of attending surgeon |

**2. What was your surgical skill level during the procedure?**

| **1** | **2** | **3** | **4** | **5** |
| --- | --- | --- | --- | --- |
| I felt I had none whatsoever |  | Average, as required of a resident my level |  | Well above average and only rarely encountered in a resident of my level |

**3. Were you worried during the procedure?**

| **1** | **2** | **3** | **4** | **5** |
| --- | --- | --- | --- | --- |
| Constantly worried something was going to go wrong (life threatening complication) or I was not operating at the required level |  | Occasionally worried |  | I felt completely calm, not worried at all |

**4. Were you anxious during the procedure?**

| **1** | **2** | **3** | **4** | **5** |
| --- | --- | --- | --- | --- |
| Constantly anxious, I had “flutters in my stomach” during the whole procedure |  | Occasionally anxious |  | Not anxious at all |

**5. Based on your performance today, would you have liked to have avoided this procedure altogether?**

| **1** | **2** | **3** | **4** | **5** |
| --- | --- | --- | --- | --- |
| Indeed |  | Only occasionally during the procedure |  | On the contrary, I would do it again anytime |

**6. How comfortable were you with the independent planning and performing of the procedure?**

| **1** | **2** | **3** | **4** | **5** |
| --- | --- | --- | --- | --- |
| Very uncomfortable |  | Average |  | Very comfortable (level of attending surgeon) |
